# Endoscopic ultrasound-guided gastroenterostomy through the mesh of a previous enteral stent in a patient with malignant gastric outlet obstruction and refractory ascites

**DOI:** 10.1055/a-2638-3031

**Published:** 2025-07-25

**Authors:** Giuseppe DellʼAnna, Francesco Vito Mandarino, Jacopo Fanizza, Gabriele Altieri, Ernesto Fasulo, Silvio Danese, Gianfranco Donatelli

**Affiliations:** 1 55727 Unité dʼEndoscopie Interventionnelle, Ramsay Santé, Hôpital Privé des Peupliers, Paris, France; 2 Gastroenterology and Gastrointestinal Endoscopy Unit, IRCCS San Raffaele Hospital, Milan, Italy; 3 27288 Gastroenterology and Gastrointestinal Endoscopy Unit, IRCCS Policlinico San Donato, Milan, Italy; 4 18985 Faculty of Medicine and Surgery, Vita-Salute San Raffaele University, Milan, Italy; 5 9307 Department of Clinical Medicine and Surgery, University of Naples “Federico II”, Naples, Italy

Endoscopic ultrasound-guided gastroenterostomy (EUS-GE) is the preferred treatment for malignant gastric outlet obstruction (mGOO) due to its minimally invasive nature and superior long-term efficacy over surgery and enteral stenting (ES). However, massive malignant ascites may constitute technical contraindications to EUS-GE 1 2 3 4 . We treated a 74-year-old man with metastatic pancreatic body adenocarcinoma infiltrating the third duodenal portion 3 months early, who underwent 22 mm × 90 mm ES (Walflex, Boston Scientific, USA) placement in another center and was referred for recurrent mGOO, which led to chemotherapy (CT) interruption. A computed tomography scan revealed massive ascites, refractory to percutaneous drainage. After a multidisciplinary discussion, EUS-GE, according to the wireless simplified technique, was proposed 5 ( Video 1 ). The endoscopic evaluation confirmed ES obstruction due to tissue ingrowth ( Fig. 1 ). During EUS, the first jejunal and adjacent loops, containing the nasogastric tube and distended by the solution, were seen floating in the ascites ( Fig. 2 ). Consequently, under EUS guidance and following the ES, an optimal window for EUS-GE was identified at its distal flange. Under EUS and fluoroscopic guidance, a 20-mm × 10-mm lumen apposing metal stent (LAMS; Hot Axios, Boston Scientific, USA) electrocautery catheter was advanced and deployed through the ES mesh, allowing the immediate intragastric flow of the blue-dyed solution ( Fig. 3 ). The patient was discharged on postoperative day 1 after restarting regular oral feeding the same day. The 2-week scheduled endoscopic control confirmed full LAMS expansion and contrast medium flow from the stomach through the LAMS to the duodenum downstream of the ES distal flange ( Fig. 4 ). After 3 months, the patient remains asymptomatic and continues CT. Although massive ascites is a contraindication to EUS-GE, this case demonstrates its feasibility in expert hands through the mesh of a previously placed ES. In a similar setting, the ES terminal portion could act as a landmark and fixation point, minimizing misdeployment risk while effectively bypassing the stenotic segment 4 .

Endoscopic ultrasound-guided gastroenterostomy through the mesh of the enteral stent.Video 1

**Fig. 1:**
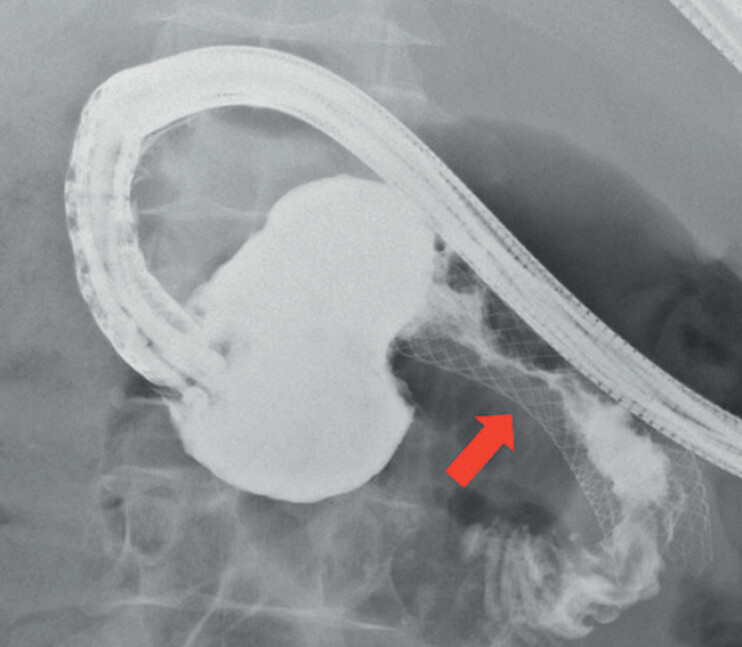
Serrated stenosis (red arrow) of the middle portion of the enteral stent due to tissue ingrowth.

**Fig. 2:**
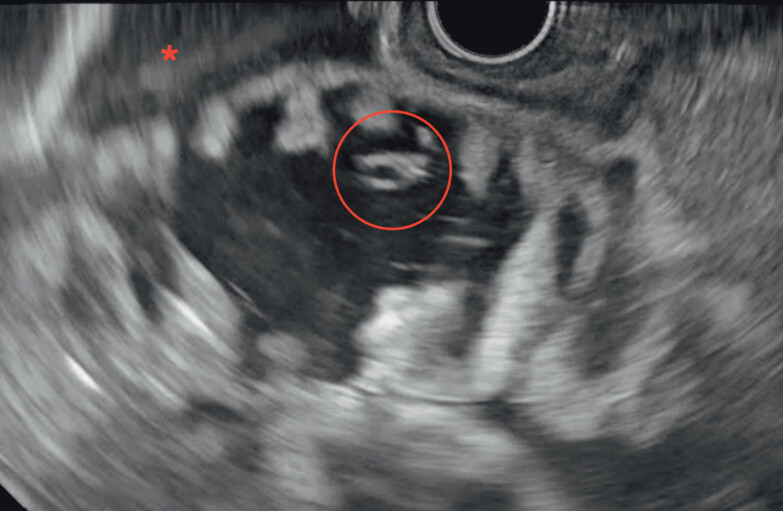
The first jejunal and adjacent loops distended with the oro-jejunal tube inside (red circle), floating in the ascites (red asterisk).

**Fig. 3:**
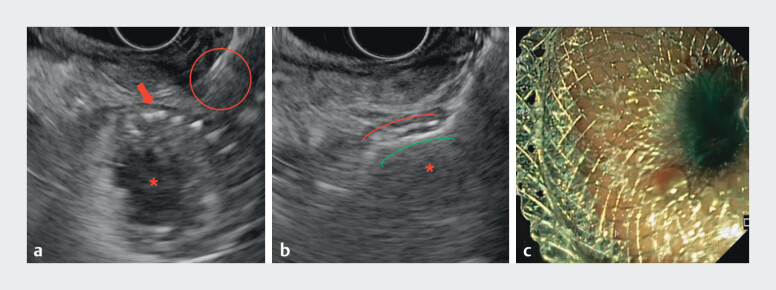
**a** Endoscopic ultrasound (EUS) view of the enteral stent (ES) lumen (red asterisk) and mesh (red flag) with the electrocautery-enhanced tip of the lumen apposing metal stent (ec-LAMS) (red circle). **b** EUS view of the release of the distal flange of the ec-LAMS (green line) inside the ES (red line) lumen (red asterisk). **c** Endoscopic view of the intragastric release of the proximal flange of the ec-LAMS with blue solution flow.

**Fig. 4:**
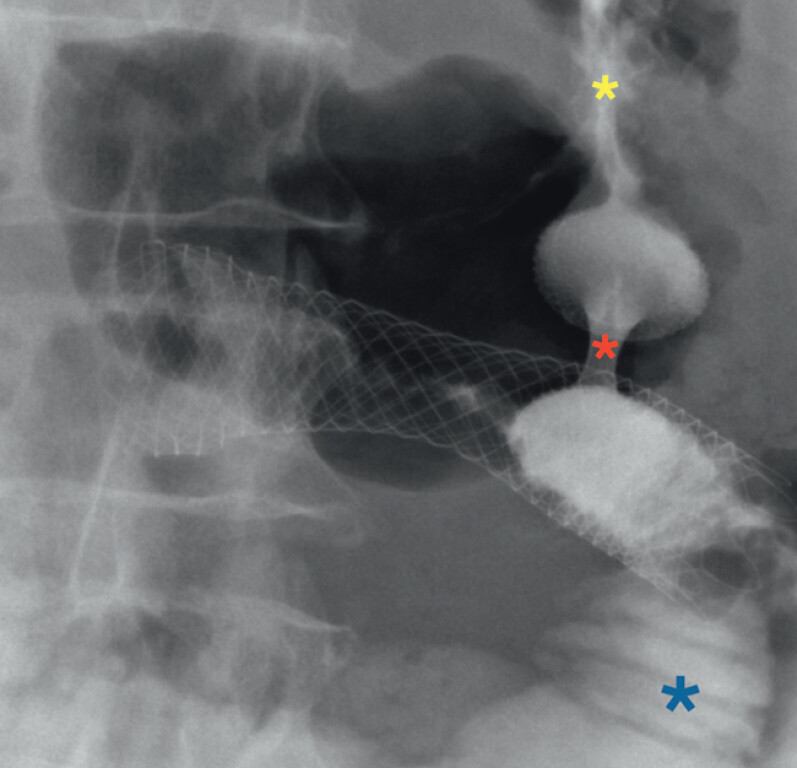
The fluoroscopic view of the contrast medium flows from the gastric lumen (yellow asterisk), through the lumen apposing metal stent lumen (red asterisk) into the first jejunal loop lumen (blue asterisk).

Endoscopy_UCTN_Code_TTT_1AS_2AK

## Notes

Endoscopy E-Videos https://eref.thieme.de/e-videos
*E-Videos* is an open access online section of the journal *Endoscopy* , reporting on interesting cases and new techniques in gastroenterological endoscopy. All papers include a high-quality video and are published with a Creative Commons CC-BY license. Endoscopy E-Videos qualify for HINARI discounts and waivers and eligibility is automatically checked during the submission process. We grant 100% waivers to articles whose corresponding authors are based in Group A countries and 50% waivers to those who are based in Group B countries as classified by Research4Life (see: https://www.research4life.org/access/eligibility/ ). This section has its own submission website at https://mc.manuscriptcentral.com/e-videos .
